# Plasma disposition of gabapentin after the intragastric administration of escalating doses to adult horses

**DOI:** 10.1111/jvim.15724

**Published:** 2020-02-08

**Authors:** Jenifer R. Gold, Tamara L. Grubb, Stephen Green, Sherry Cox, Nicolas F. Villarino

**Affiliations:** ^1^ Department of Veterinary Clinical Sciences Washington State University Pullman Washington; ^2^ Department of Biomedical and Diagnostic Sciences University of Tennessee Knoxville Tennessee

**Keywords:** analgesia, equine, laminitis, neuropathic pain, pharmacokinetics

## Abstract

**Background:**

In humans, gabapentin an analgesic, undergoes non‐proportional pharmacokinetics which can alter efficacy. No information exists on the pharmacokinetics of dosages >20 mg/kg, escalating dosages or dose proportionality of gabapentin in horses.

**Hypothesis and Objectives:**

Gabapentin exposure in plasma would not increase proportionally relative to the dose in horses receiving dosages ≥20 mg/kg. To assess the plasma pharmacokinetics of gabapentin after nasogastric administration of gabapentin at dosages of 10 to 160 mg/kg in adult horses.

**Animals:**

Nine clinically healthy adult Arabian and Quarter Horses.

**Methods:**

In a randomized blinded trial, gabapentin was administered by nasogastric intubation to horses at 10, 20 mg/kg (n = 3) and 60, 80, 120, 160 mg/kg (n = 6). Plasma was collected before and at regular times over 64 hours after administration of gabapentin. Gabapentin was quantified using a validated chromatographic method. Dose proportionality was estimated using a power model. Pharmacokinetic parameters were estimated using compartmental pharmacokinetic analysis.

**Results:**

Plasma pharmacokinetics parameters of gabapentin were estimated after nasogastric administration at dosages of 10 to 160 mg/kg. Gabapentin plasma concentration increased with dose increments. However, the area under the concentration curve from zero to infinity and maximal plasma concentration did not increase proportionally relative to the dose in horses.

**Conclusions and Clinical Importance:**

Gabapentin exposure in plasma is not proportional relative to the dose in horses receiving nasogastric dosages of 10 to 160 mg/kg.

**Abbreviations**
AUC: area under the curve
C_max_: maximum concentration
FDA: Food and Drug Administration

## 1 INTRODUCTION

Gabapentin (1‐[aminomethyl] cyclohexane acetic acid) was first approved by the Food and Drug Administration (FDA) in 1993^1^ as an anticonvulsant in humans and is currently used to control seizures in both humans and animals.^2^, ^3^, ^4^ Gabapentin also plays an important role in pain management and was FDA‐approved for treatment of humans with post‐herpetic neuralgic pain, a type of neuropathic pain, in 2002.^5^, ^6^

Laminitis is an extremely painful, debilitating disease of horses, which is devastating to the animals and the animal owners because of the inability to control the pain in horse and the high financial cost/loss. Neuropathic changes typical of those described in other neuropathic pain syndromes are present in horses with laminitis.^7^ Neuropathic pain is often unresponsive to traditional analgesic treatment because of damage to and changes in a variety of the components in the pain pathway resulting in amplification and expansion of pain signals. Gabapentin is 1 of the few drugs that controls some forms of neuropathic pain in humans.^8^ Although there is a paucity of information on the use of gabapentin in species other than humans, it has been administered to control neuropathic pain in small animals; however, dosing, and likely the type of disease, also complicates gabapentin efficacy in these dogs and cats.^9^, ^10^, ^11^, ^12^, ^13^, ^14^, ^15^, ^16^ Failure to prove efficacy is potentially the result of inadequate dosing.^17^, ^18^

Gabapentin is most commonly used to control the pain of laminitis and other conditions that cause neuropathic pain at 5‐20 mg/kg twice daily^19^, ^20^ in combination with nonsteroidal anti‐inflammatory agents.^18^, ^21^, ^22^, ^23^ Treatment has not always been successful but the dosage regimens have been extrapolated from humans and efficacy of gabapentin with appropriate doses remains unknown. In order to determine efficacy of the drug, a dosing regimen that could potentially provide analgesia must be established.

After oral administration to horses, gabapentin is rapidly absorbed but the extent of absorption is relatively poor (mean oral bioavailability of gabapentin [±SD] was 16.2% ± 2.8%),^19^ meaning that higher dosages might be required to achieve therapeutic plasma concentrations. In addition, in humans, gabapentin undergoes saturation pharmacokinetics so that the serum concentration of the drug is nonlinear to the dose administered (dose disproportionality).^24^, ^25^ Horses have an intestinal transporter similar to humans, thus dose disproportionality and nonlinear pharmacokinetics might occur.^26^ Drugs with nonlinear kinetics limit the possibility of predicting changes in drug exposure when dosages regimens are adjusted. Because neuropathic pain can be refractory in many conditions and the extent of pain is highly variable among patients, individualization of treatments and adjustment of dosage regimens are often required for control of individual analgesic needs. However, it remains to be determined if higher doses of gabapentin result in higher plasma exposure after enteric administration. The adverse effects of gabapentin administered at >20 mg/kg to horses are undetermined. In humans, somnolence dizziness and ataxia are frequent adverse effects of gabapentin.^1^ Sedation and ataxia occurs in dogs and cats receiving gabapentin.^12^, ^14^

Our main hypothesis is that doses of gabapentin between 10 and 160 mg/kg administered via nasogastric tube to horses will not increase the plasma concentration in a proportional manner. We addressed this hypothesis by evaluating the plasma pharmacokinetics of gabapentin in horses after a single nasogastric administration at 10, 20, 40, 60, 80, 120, and 160 mg/kg.

## 2 MATERIALS AND METHODS

The study was approved by the Washington State University's Institutional Animal Care and Use Committee. In this blinded randomized experimental study, 9 clinically healthy adult horses were used. The horses were 2 Quarter Horse cross mares and 7 Arabian mares. The mean weight of the horses was 444 ± 15.68 kg. The equine age ranged from 8‐23 years with a mean age of 13.2 years.

The horses were housed together on a dry lot with free choice water, selenium/mineral salt and were fed grass hay twice daily when they were not involved with the research project. While the study was being performed, the horses were brought into the WSU teaching facility stalls 24‐48 hours before the first treatment and were fed a diet of grass hay daily and free choice water. The horses were held off feed the morning of the research project and were fed their standard portion of hay, 3 hours after gabapentin administration. Water was available at all times. The afternoon before the gabapentin administration, each horse had a jugular vein catheter placed aseptically. The catheter was then heparin‐locked (2.5 mL normal saline, 2.5 mL of 100 units/mL heparin). The horses were evaluated at that time for any signs of lameness or other health concerns. The study began at 5:30 am with physical examinations and confirmation of the absence of ataxia or sedation. The horses were sedated with xylazine (AnaSed 100 mg/kg, Santa Cruz Animal Health, Dallas, Texas; 0.5‐1.0 mL/kg) before the passage of a nasogastric tube. Gabapentin (Gabapentin 600 mg tablets Blue Point Labs, Egypt; 600 mg) tablets were ground in a coffee grinder, and all doses were reconstituted with 250 mL of water to maintain blinding of evaluators. The gabapentin/water mixture was gravity fed through a funnel into a nasogastric tube to ensure that the entire dose was delivered. The nasogastric tube was flushed with 1 L of water and then removed. Sedation was reversed with atipamezole hydrochloride (Antisedan 5 mg/mL, Zoetis Inc, Kalamazoo, Michigan; 0.5‐1.0 mg/kg to effect). Horses were randomly allocated to each dose level using a random number generator program (http://random.org, Dublin, Ireland). Due to horse availability, this was a modified cross‐over study with 3 horses receiving doses of 10, 20, and 40 mg/kg gabapentin. Six different horses received all 4 doses of 60, 80, 120, and 160 mg/kg of gabapentin in a cross‐over design. All horses had a 2‐week washout period in between doses, where they were placed back out into a dry lot with other horses.

Plasma samples were collected via the catheter in heparinized tubes pre‐dose and time, 0.25, 0.50, 1, 2, 4, 8, 16, 32, and 64 hours post‐administration of gabapentin. Initially, 5 mL of blood were drawn through the catheter and discarded, then a 5 mL sample of blood was collected and injected into a heparinized tube, finally the catheter was flushed with 50 USP units of heparin/5 mL normal saline. The blood was centrifuged at 1800*g* for 5 minutes. Plasma was stored at −80°C until analysis.

At each sampling time, 2 blinded evaluators (the same 2 throughout the study) completed a full physical examination which included heart rate, respiratory rate, body temperature, mucous membrane color, capillary refill time, gastrointestinal sounds, and observation of or the presence of feces and urination of each horse. In addition, a sedation score was assigned using a sedation scoring system.^20^, ^27^ The scoring system used was: 0—No sedation (normal movement, normal ear and neck position, normal posture); 1—Mild sedation (slightly decreased frequency and rapidity of movement, lowered ear and neck, lip drooping, slightly relaxed postural tone); (2)—Moderate sedation (moderately decreased frequency and rapidity of movement, ear tip separation, neck position below the horizontal plane); 3—Deep sedation (prolonged periods of immobility, pronounced ear tip separation, loss of postural tone, base wide stance). To ensure that sedation was identified if it occurred, the horses were continuously observed for the first 4 hours after gabapentin administration and a sedation score was assigned at every data collection time point throughout the entire study. The equine response to a loud clapping noise was done to help determine sedation level. The horses were also walked in a tight circle and straight line to look for any signs of ataxia. The Mayhew ataxia scale was utilized; grade 0, normal; grade 1, subtle gait abnormality that may get worse with head elevation. grade 2, moderate gait abnormalities noted at a walk; grade 3, easily recognizable gait abnormalities that are much worse when animal is going around obstacles or head is elevated; grade 4, easily seen gait abnormalities with the potential that the horse will fall easily or nearly fall when asked to walk or perform normal activities; grade 5, recumbent horse.^28^

### 2.1 Quantification of gabapentin in plasma from horses—High‐performance liquid chromatography

Gabapentin was extracted from plasma samples using the pre‐column derivatization, solid‐phase extraction method of Mercolini et al.^29^ Previously frozen plasma samples were thawed and vortex‐mixed, and 100 μL were transferred to a clean tube, then 30 μL of internal standard (vigabatrin 10 μg/mL) added followed by 1 mL of 0.1 N HCL. This mixture was loaded onto a preconditioned MCX cartridge (Waters MCX cartridge, Waters Corporation, Milford, Massachusetts). Samples were eluted with 2 mL of ammonia:water:acetonitrile (5:13:82) and evaporated to dryness with nitrogen gas.

Analysis of gabapentin in plasma samples was conducted using reversed‐phase high‐performance liquid chromatography. The system consisted of a 2695 separations module and a 2475 fluorescence detector (Waters). Separation was attained on a Waters Atlantis T3 4.6 × 250 mm (5 μm) preceded by a 5‐μm Atlantis T3 guard column. The mobile phase was a mixture of (A) 50 mM potassium phosphate dibasic buffer (pH 5.0) and (B) acetonitrile. Gradient elution was used to separate the analytes starting with 53% of solution A and 47% of solution B and was adjusted to 49% of solution A and 51% of solution B over 15 minutes, and back to initial conditions over 5 minutes. The flow rate was 1.1 mL/min. The fluorescence detector was set at an excitation of 300 and an emission of 500 with the gain at 10×. The column was at ambient temperature.

Standard curves for plasma analysis were prepared by spiking untreated plasma with gabapentin, which produced a linear concentration range of 25‐10 000 ng/mL. Average recovery was 87% for gabapentin. Intra‐assay variability ranged from 1.0% to 5.4%, whereas inter‐assay variability ranged from 0.4% to 11% for gabapentin, respectively. The lower limit of quantification was 25 ng/mL.

### 2.2 Estimation of pharmacokinetic parameters

Compartmental analysis was used to calculate primary and secondary pharmacokinetic parameters as implemented by Phoenix WinNonlin v8.0 (Phoenix WinNonlin v8.0f, Pharsight Corp, Mountain View, California). Pharmacokinetic parameters included area under the plasma concentration‐time curve from 0 hours to infinity after dosing (area under the curve, AUC_0‐∞_) maximum concentration (Cmax), time to maximum concentration (Tmax), absorption rate constant (K01), half‐life of absorption K01_HL), terminal rate constant (K10), half‐life of terminal portion of the curve after oral administration (K10_HL), microdistribution rate constant from the central compartment (1) to peripheral compartment (2) (k12), and from the peripheral (2) to central compartment (1) (k21).

For compartmental analysis, we implemented a standard 2‐stage approach in order to select the simplest model that fits the observed concentration data.^30^, ^31^, ^32^, ^33^ Plasma drug concentrations were plotted on linear and semi‐logarithmic graphs for analysis and to allow visual assessment of the best model for pharmacokinetic analysis. We defined the compartment structure implementing standard procedures and diagnostic tools (standard errors of the estimates, correlation matrix, and residual plots, F test, Akaike's information criterion, and Schwarz criteria), in order to select the simplest model that fits the experimental data. Compartmental analysis of results for each dose level was finally performed by weighting the values by 1/*y* ^2^, where *y* is the predicted concentration at each time. A 1‐or 2‐compartmental model with first‐order absorption was used with corresponding equations of

$$C\;\left(T\right)=\mathrm{DK}01/V\left(K01-K10\right)\;\left[\exp\left(-K10\times T\right)-\exp\left(-K01\times T\right)\right]$$

(1‐compartment model, first‐order absorption, no lag time)

$$C\;\left(T\right)=A\;\exp\left(-\alpha \times T\right)+B\;\exp\left(-\beta \times T\right)+C\;\exp\left(-K01\times T\right)$$

(2‐compartments model, first‐order absorption, no lag time).where K01 is the absorption rate post‐nasogastric administration, assuming first‐order absorption; *D* is the oral dose; *V* is the apparent volume of distribution; and, K10 is the elimination rate constant.

### 2.3 Statistical analysis

The median AUC_0‐∞_ for each dose level was compared statistically using Kruskall‐Wallis test using Prism 3.03 GraphPad software (La Jolla, California). The level of significance was set at *P* < .05.

No statistically significant findings were noted in any of the physical examination variables, ataxia, or sedation score of the horses.

### 2.4 Plasma concentration of gabapentin following simulated multiple dose administrations

For the simulation of plasma gabapentin concentrations after multidose drug administration, data from each horse and dose level (10, 20, 60, 80, 120, 160 mg/kg) was modeled using compartmental analysis and the modeling procedures described above. The mean pharmacokinetics data was used to simulate plasma concentrations of gabapentin at each dose level and 3 dosing intervals scenarios: every 8, 12, and 24 hours over a period of 120 hours.

### 2.5 Determination of dose proportionality and statistical analysis

The dose proportionality was tested using a standard method known as power model approach^31^

$$\mathrm{Ln}\;\left({\mathrm{AUC}}_{0-\infty \text{or}}\;{\text{Cmax}}_{\mathrm{ij}}\right)=B_{0}+\eta_{1}+\left(B_{1}+\eta_{2i}\right)\times \mathrm{Ln}\;\left({\text{dose}}_{\mathrm{ij}}\right)+\varepsilon_{\mathrm{ij}},$$

where Ln is the Napierian logarithm, AUC_0‐last_, and Cmax are the dependent variable *j*th observation in the *i*th subject and dose the independent variable, *η*_1_ the random intercept (*B*_0_) and *η*_2_ the random slope (*B*_1_) and error ε_*ij*._ Dose proportionality would be declared when the (1 − *α*) × 100 confidence interval (CI) of the slope lies entirely within the critical region:

$$\left(1+\mathrm{Ln}\;\left(\theta_{\mathrm{low}}\right)/\ln\left(r\right),1+\mathrm{Ln}\;\left(\theta_{\text{high}}\right)/\mathrm{Ln}\left(r\right)\right)$$

Where Ln is the Napierian logarithm, θ_high (_160 mg/kg), and θ_low_ (10 mg/kg) are the preestablished limits of the CI at the lower (0.80) and high ends (1.25), respectively. The advantage of this approach is that it not only tests dose proportionality but also provides evidence of the degree disproportionality.^30^, ^32^, ^33^

## 3 RESULTS

### 3.1 Physical examination variables and sedation scores

There were no differences among dosage levels in heart rates, respiratory rates, and body temperatures and all remained within normal limits. Subjectively, appetite, urine, and fecal production/consistency did not change. Sedation was identified in 1 horse at exactly 2 hours after administration of 120 and 160 mg/kg of gabapentin. The highest sedation score was 2 but returned to 0 after 1 hour. All other sedation scores and all ataxia scores remained 0 throughout the study.

### 3.2 Estimation of pharmacokinetic parameters and simulated plasma concentration of gabapentin following multiple dose administrations

**Figure 1:**
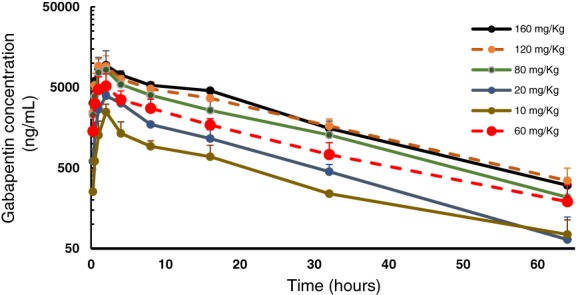
Plasma concentration (mean ± SD) versus time profile of gabapentin following the nasogastric administration of gabapentin at 10 (n = 3), 20 (n = 3), 60 (n = 3), 80 (n = 6), 120 (n = 6), and 160 (n = 6), mg/kg to adult horses

**Figure 2:**
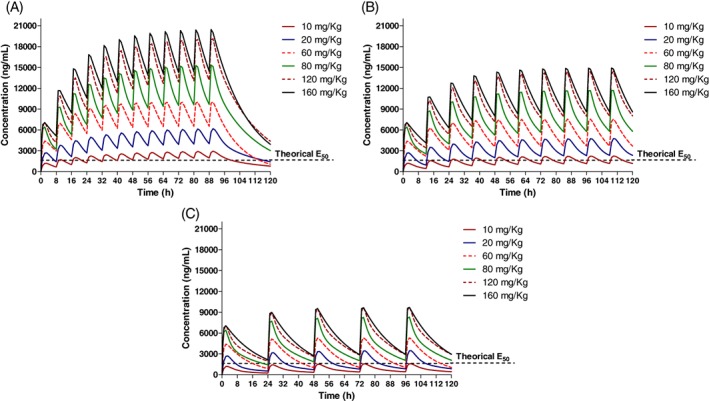
Simulated plasma disposition of gabapentin in horses for different dose levels after the oral administration every 8 (A), 12 (B), and 24 hours (C)

Following the nasogastric administration of gabapentin at all doses, the drug was detected in all horses (Figure 1). Pharmacokinetic parameters were determined by compartmental analysis are presented in Table 1. For all dose levels and horses, the percent AUC extrapolated to infinity was <17%. Simulated plasma concentration of gabapentin following multiple dose administrations are displayed in Figure 2. The median AUC_0‐∞_ after the administration of gabapentin at 10 mg/kg was lower than the median AUC_0‐∞_ obtained after the administration of gabapentin at 120 (*P* = .02) and 160 mg/kg (*P* = .008).

**Table 1:** Plasma pharmacokinetic parameters (median [range]) derived from a 1‐ or 2‐compartmental model with first‐order absorption for gabapentin in horses after a single oral administration at 10, 20, 80, 120, and 160 mg/kg of body weight

Dose (mg/kg)	Pharmacokinetics parameters								
	K01 1/h	K01_HL h	K10 1/h	K10_HL h	k12 1/h	k21 1/h	Tmax h	Cmax μg/mL	AUC_0‐∞ μg × h/mL_
10 (n = 3)	0.6 (0.478‐0.760)	1.1 (0.9‐1.5)	0.13 (0.06‐0.18)	5.5 (3.9‐11.3)	0.2 0.02‐0.34	0.10 (0.03‐0.16)	2.6 (2‐3.3)	1.6 (1.5‐2.4)	31^a^ (27–35)
20 (n = 3)	0.9 (0.467‐1.09)	0.7 (0.6‐1.5)	0.09 (0.08‐0.11)	7.6 (6.1‐8.2)	0.21 (NA)	0.18 (NA)	2.7 (2.5‐2.9)	3.2 (3.2‐3.5)	49 (48‐61)
60 (n = 6)	1.5 (0.747‐2.07)	0.5 (0.3‐0.9)	0.07 (0.05‐0.11)	7.2 (2.1‐13.8)	0.2 (0.001‐0.52)	0.2 (0.3‐0.43)	1.9 (1.5‐2.4)	4.0 (2.8‐8.4)	79 (52‐150)
80 (n = 6)	0.8 (0.506‐2.818)	0.9 (0.2‐1.4)	0.12 (0.06‐0.17)	6.0 (4.1‐11.9)	0.24 (0.2‐0.41)	0.2 (0.14‐0.56)	2.2 (1.0‐2.7)	6.7 (3.2‐10)	110 (69‐180)
120 (n = 6)	0.8 (0.792‐1.81)	0.8 (0.5‐2.1)	0.09 0.04‐0.05	7.9 (13.3‐15.7)	0.45 (0.40‐0.6)	0.3 (0.2‐0.4)	1.8 (1.4‐3.7)	8.4 (4.8‐15)	150^b^ (120‐220)
160 (n = 6)	2.3 (0.522‐3.492)	0.3 (0.1‐1.3)	0.06 (0.05‐0.12)	11.5 (4.1‐13.9)	0.39 (0.26‐0.52)	0.3 (0.3–0.4)	1.4 (1.0‐2.6)	8.5 (5‐11)	160^b^ (110‐230)

*Note*: AUC_0‐∞_, area under the plasma concentration‐time curve from 0 hours to infinity after dosing; Cmax, maximum concentration; K01, absorption rate constant; K01_HL, half‐life of absorption; K10, terminal rate constant; K10_HL, half‐life of terminal portion of the curve after oral administration; k12 and k21, microdistribution rate constants from the central compartment (1) to peripheral compartment (2) and from the peripheral (2) to central compartment (1), respectively; NA, not applicable; Tmax, time to maximum concentration. The median AUC_0‐∞_ for each dose level was compared statistically using Kruskall‐Wallis and Dunn's multicomparison tests. The different superscript letters indicate *P* < .05.

### 3.3 Determination of dose proportionality

**Figure 3:**
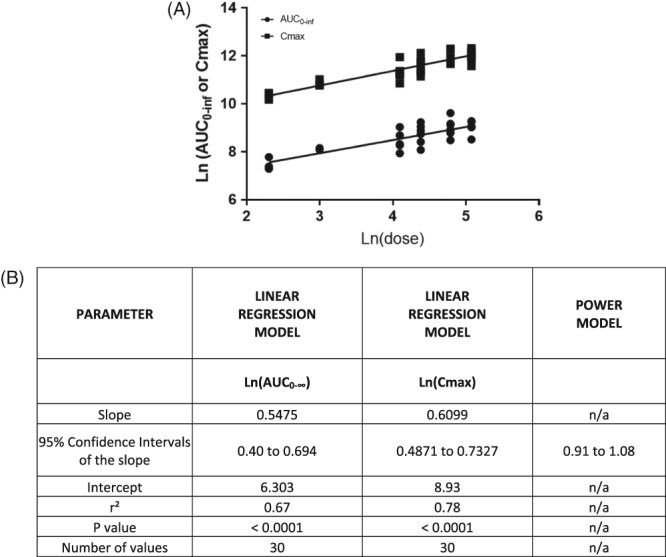
Linear log‐log regression. A, AUC_last_ (derived from compartmental analysis) and Cmax corresponding to doses ranging from 10 to 160 mg/kg. B, Corresponded to linear regression and power model parameter values. AUC, area under the curve; Cmax, maximum concentration

Gabapentin plasma concentrations increased across dose levels with no proportional change with dose escalation for either AUC_0‐∞_ or C_max_. The a priori CI (derived from the power model) for the tested gabapentin dose ratio (160/10 = 16) was 0.90‐1.09. The 95% CI for the slope derived from the regression model was 0.40 to 0.694 and 0.4871 to 0.7327 for AUC_0‐∞_ and Cmax respectively (Figure 3D). Therefore, there is no evidence that supports that the changes in drug exposure are proportional to the dose (Figure 3).

## 4 DISCUSSION

Plasma pharmacokinetics of gabapentin administered via nasogastric tube to horses at single doses ranging from 10‐160 mg/kg are reported here. Plasma terminal half‐life of gabapentin ranged from 2 to 15.7 hours (Table 1). This is in contrast to the results reported by 3.4 and 7.7 hours (6.7‐11.9 hours) after the oral administration of 5 and 20 mg/kg, respectively.^19^, ^20^ The reason(s) for these discrepancies are unclear. As we hypothesized, gabapentin plasma AUC_0‐∞_ and Cmax increased (Figure 3) across dose levels with no evidence to support that the increments in drug exposure are proportional in magnitude to the dose increment, as occurs in humans^5^ and dogs.^12^, ^13^ In fact, the results suggest that plasma concentration of gabapentin increases in a less than proportional manner. The lack of dose proportionality in plasma concentration of gabapentin could be a result of saturation of intestinal transporters and a relative decrease in intestinal absorption. The presence of food in the stomach also impacts drug uptake, including uptake via active transport systems and could have impacted our results^34^ A standardized feeding regimen might be an important component of eventual pharmacodynamic efficacy studies. The feeding regimens in other studies of gabapentin in horses were not reported.

There is no information that describes the optimum analgesic plasma concentrations for gabapentin in horses. In humans, half maximal effective concentration (EC50) for treating neuropathic pain was 5.4 μg/mL.^5^, ^35^ Studies in rats suggest that half maximal effective concentration (EC50) range from 1.4 and 16.7 μg/mL for treatment of hyperalgesia.^5^ Simulated plasma concentrations of gabapentin (Figure 2) suggest that all the dosage regimens would attain 16.7 μg/mL (represented as the theoretical EC50 in Figure 3) relatively quickly following the administration of gabapentin. Furthermore, the administration of gabapentin at ≥10 mg/kg every 8 hours, ≥20 mg/kg every 12 hours or ≥80 mg/kg every 24 hours would maintain plasma concentrations above 16.7 μg/mL during the dose interval. However, there is no evidence to support that the efficacious concentrations reported for rats would apply to horses. Further pharmacokinetic and pharmacodynamic modeling would help to elucidate effective and safe gabapentin dosage regimens for treating horses. One of the current barriers for establishing an optimal dosage regimen is the lack of validated neuropathic pain models in horses.

Based on the data generated in this study, the use of 160 mg/kg of gabapentin would not provide a relevant higher exposure compared to the administration of 120 mg/kg. Hence, it is unlikely that doses higher than 120 mg/kg would provide any noticeable differences in the pharmacological effect.

No adverse effects occurred except mild sedation at 120 and 160 mg/kg of gabapentin in 1 horse. Therefore, it appears that gabapentin is tolerable at doses higher than those currently recommended in clinical practice. However, a limitation is that this study was not designed to assess the safety of the doses used. Gabapentin‐induced hepatocellular injury^4^, ^36^ should be considered in future research involving multidose and long‐term gabapentin administration. Gabapentin‐related chronic kidney disease occurs in humans.^37^, ^38^ Since gabapentin is eliminated by the kidneys, monitoring renal function in horses receiving multiple doses of gabapentin would be important, particularly in horses concurrently receiving NSAIDs,^39^, ^40^ which can also cause renal damage, for treatment of laminitis or other neuropathic pain.

The fact that horses were sedated with xylazine to facilitate passage of the nasogastric tube to deliver gabapentin is a potential limitation as xylazine slows intestinal motility,^41^ which could alter the uptake of orally administered drugs. However, the effects of the xylazine were antagonized with atipamazole, which is shown to return intestinal motility to normal.^41^ Clinically, all horses had normal gastrointestinal borborygmi and fecal production, subjectively indicating normal motility. All horses received xylazine/atipamazole, thus any potential impact would be uniform across all dosages.

Another limitation is the fact that the pharmacokinetics of gabapentin following a single‐dose administration was assessed in a relatively small number of adult horses and therefore these findings should be confirmed with a larger population of animals receiving multiple dosages, as would occur in clinical treatment of chronic pain. Multiple dosage regimen studies would provide valuable information about potential adverse effects or toxicoses not detected in our study, such as previously mentioned hepatic and renal dysfunction^36^, ^37^, ^38^ (Figure 2). Results of this study suggest that gabapentin might accumulate in plasma if it is administered at a <30‐hour dosing interval until steady state is reached.

This study did not include IV administration of gabapentin. A commercially available injectable solution was not available at the time and thus bioavailability and IV pharmacokinetics could not be performed. Finally, no pharmacodynamic assessment was performed at varying dosages; therefore, there is no ability to recommend a dose to treat neuropathic pain based on our results.

In conclusion, this study reports the disposition of gabapentin after the administration of multiple escalating doses. However, safety and efficacy of repeated dose administration higher than 20 mg/kg have yet to be confirmed.

## CONFLICT OF INTEREST DECLARATION

Authors declare no conflict of interest.

## OFF‐LABEL ANTIMICROBIAL DECLARATION

Authors declare no off‐label use of antimicrobials.

## INSTITUTIONAL ANIMAL CARE AND USE COMMITTEE (IACUC) OR OTHER APPROVAL DECLARATION

Approval by the Washington State University IACUC, ASAF#4877.

## HUMAN ETHICS APPROVAL DECLARATION

Authors declare human ethics approval was not needed for this study.
